# Preventable tragedies: findings from the #NotAnAccident index of unintentional shootings by children

**DOI:** 10.1186/s40621-023-00464-3

**Published:** 2023-10-23

**Authors:** Ashley D. Cannon, Kate Reese, Paige Tetens, Kathryn R. Fingar

**Affiliations:** Everytown for Gun Safety Support Fund, PO Box 4184, New York, NY 10163 USA

**Keywords:** Unintentional injuries, Firearm, Children, Unintentional gun deaths, Gun accidents, Injury surveillance, Child access prevention, Firearm storage

## Abstract

**Background:**

Between 2015 and 2021, 3,498 Americans died from unintentional gun injuries, including 713 children 17 years and younger. Roughly 30 million American children live in homes with firearms, many of which are loaded and unlocked. This study assesses the scope of unintentional shootings by children 17 and younger in the US and the relationship between these shootings and state-level secure storage laws.

**Methods:**

Demographic and injury data of both perpetrators and victims of unintentional shootings by children 17 and younger in the US from 1/1/2015–12/31/2021 were extracted from the #NotAnAccident Index. The #NotAnAccident Index contains media-report data, which is systematically flagged through Google Alerts. We describe characteristics of incidents and examine incident rates over time. The association between state-level secure storage laws and rates of unintentional shootings by children is assessed in multivariate negative binomial regression models.

**Results:**

2,448 unintentional shootings by children resulted in 926 deaths and 1,603 nonfatal gun injuries over a period of seven years. Most perpetrators (81%) and victims (76%) were male. The mean age was 10.0 (SD 5.5) for shooters and 10.9 (SD 8.1) for victims. Children were as likely to shoot themselves (49%) as they were to shoot others (47%). The majority of victims were under 18 years old (91%). Shootings most often occurred in or around homes (71%) and with handguns (53%). From March to December 2020, coinciding with the COVID-19 pandemic, incidents increased 24% over the same period in 2019, which was driven largely by an increase among shooters ages 0–5. Depending on the type of law, rates of unintentional shootings by children were 24% to 72% lower in states with secure storage laws, compared to states without such laws.

**Conclusions:**

Unintentional shootings by children are on the rise, particularly among children 0–5 years old, but are preventable tragedies. Our results show that secure firearm storage policies are strongly correlated with lower rates of unintentional shootings by children. Firearm storage policies, practices, and education efforts are needed to ensure guns are kept secured and inaccessible to children.

## Background

Centers for Disease Control and Prevention (CDC) Vital Statistics show that between 2015 and 2021, 3498 Americans died from unintentional gun injuries, including 713 children 17 years and younger (Centers for Disease Control and Prevention 2021, 2023). While the rate of unintentional gun deaths increased 8.7% among all ages in 2021, compared to 2015, the rate among children under 18 years old increased 82.0% during the same period (Centers for Disease Control and Prevention 2021, 2023). These numbers are believed to be an underestimate as unintentional deaths are often misclassified in Vital Statistics as homicides, particularly for deaths of children (Schaechter et al. 2003; Barber and Hemenway 2011; Luo and McIntire 2013; Hemenway and Solnick 2015; Solnick and Hemenway 2019).

The number of children living in homes with firearms has grown in recent years. Researchers estimate that roughly 30 million American children live in homes with firearms—up 7 million since 2015 (Miller and Azrael 2022). Despite research showing that securely storing firearms (i.e., unloaded, locked, and separate from ammunition) can significantly reduce the risks of gun injuries (Grossman et al. 2005; Monuteaux et al. 2019; Violano et al. 2018), an estimated 54% of gun owners do not store their firearms securely (Crifasi et al. 2018). Roughly 4.6 million children live in households with at least one loaded and unlocked firearm (Miller and Azrael 2022). Although many gun owners believe that their children cannot access their firearms, many children know where guns are stored and can quickly access them (Baxley and Miller 2006; Simonetti et al. 2015; Salhi et al. 2021).

In recent years, there has been a growing body of research examining unintentional gun deaths most commonly utilizing the National Violent Death Reporting System (NVDRS) (Barber and Hemenway 2011; Hemenway and Solnick 2015; Solnick and Hemenway 2019) or the National Fatality Review Case Reporting System (NFR-CRS) (Trigylidas et al. 2021; Trigylidas et al. 2019). However, these studies are subject to some limitations. First, these datasets are limited to participating states. Second, while it is possible to extract data on the perpetrators of these shootings from the narratives provided in these records, these datasets are victim-centric and only include incidents resulting in a fatality.

Firearms have surpassed motor vehicles as the leading cause of death among children under 18 years old, (Centers for Disease Control and Prevention 2023b) making it more urgent to identify opportunities for prevention. This study utilizes Everytown for Gun Safety Support Fund’s (Everytown) #NotAnAccident Index, a perpetrator-centric dataset of unintentional shootings by children 17 and younger that result in gunshot injury or death having occurred in any of the 50 states and Washington, DC (Everytown for Gun Safety Support Fund 2023). Only one study that we are aware of has utilized this dataset, but limited its analysis to nonfatal and fatal firearm injuries among children ages 1–6 (Bleyer et al. 2021).

This study sought to assess the scope of unintentional shootings by children 17 years and younger in the United States to determine if differences exist by gender, age, victim type, location, weapon, state, and state-level secure storage laws.

## Results

### Characteristics of unintentional shootings by children

Over the seven years from January 2015 to December 2021, the #NotAnAccident Index recorded 2448 incidents of a child under the age of 18 unintentionally shooting themself or another person in the US (Table 1). These 2448 incidents resulted in 926 people shot and killed (0.38 average per incident) and 1603 people shot and wounded (0.65 average per incident) over the study period. The number of victims per incident ranged from one to three, with the overwhelming majority of incidents resulting in one victim (one victim, 96.8%; two victims, 3.1%; three victims, 0.1%).

**Table 1:** Characteristics of unintentional shooting incidents by children, 2015–2021

Characteristic	Total		Shooter’s age								Shooter’s gender					
			0–5		6–13		14–17		Unknown		Male		Female		Unknown	
	N	%	N	%	N	%	N	%	N	%	N	%	N	%	N	%
Total incidents	2448	100.0	763	31.2	596	24.3	894	36.5	195	8.0	1993	81.4	202	8.3	253	10.3
Total victims and mean number per incident	2529	1.03	784	1.03	610	1.02	923	1.03	212	1.09^a^	2044	1.03	215	1.06^a^	270	1.07^a^
Killed	926	0.38	287	0.38	228	0.38	356	0.40	55	0.28^a^	776	0.39	69	0.34	81	0.32^a^
Wounded	1603	0.65	497	0.65	382	0.64	567	0.63	157	0.81^a^	1268	0.64	146	0.72^a^	189	0.75^a^
*Location*																
Home	1749	71.4	629	82.4	469	78.7	528	59.1^b^	123	63.1^b^	1445	72.5	152	75.2	152	60.1^b^
Car	193	7.9	78	10.2	33	5.5^b^	71	7.9	11	5.6^b^	156	7.8	17	8.4	20	7.9
Public place	138	5.6	18	2.4	21	3.5	89	10.0^b^	10	5.1^b^	116	5.8	9	4.5	13	5.1
Hunting, target shooting	82	3.3	2	0.3	31	5.2^b^	43	4.8^b^	6	3.1^b^	70	3.5	6	3.0	6	2.4
Other^c^	28	1.1	6	0.8	9	1.5	12	1.3	1	0.5	22	1.1	2	1.0	4	1.6
Unknown	258	10.5	30	3.9	33	5.5	151	16.9^b^	44	22.6^b^	184	9.2	16	7.9	58	22.9^b^
*Type of gun*																
Handgun	1298	53.0	485	63.6	308	51.7^b^	445	49.8^b^	60	30.8^b^	1129	56.6	107	53.0	62	24.5^b^
Rifle	108	4.4	11	1.4	46	7.7^b^	42	4.7^b^	9	4.6^b^	92	4.6	5	2.5	11	4.3
Shotgun	99	4.0	8	1.0	38	6.4^b^	45	5.0^b^	8	4.1^b^	84	4.2	6	3.0	9	3.6
Other^d^	17	0.7	3	0.4	6	1.0	7	0.8	1	0.5	11	0.6	5	2.5^b^	1	0.4
Unknown	926	37.8	256	33.6	198	33.2	355	39.7^b^	117	60.0^b^	677	34.0	79	39.1	170	67.2^b^
*Shooting type*																
Shot themselves	1195	48.8	562	73.7	258	43.3^b^	350	39.1^b^	25	12.8^b^	1026	51.5	124	61.4^b^	45	17.8^b^
Shot someone else	1156	47.2	183	24.0	326	54.7^b^	521	58.3^b^	126	64.6^b^	922	46.3	69	34.2^b^	165	65.2^b^
Shot themselves and someone else	44	1.8	15	2.0	7	1.2	16	1.8	6	3.1	30	1.5	7	3.5^b^	7	2.8
Unknown	53	2.2	3	0.4	5	0.8	7	0.8	38	19.5^b^	15	0.8	2	1.0	36	14.2^b^
*Victim’s age years*^e^																
Mean and SD	10.9	8.1	5.4	7.3	10.6^a^	7.1	15.8^a^	5.5	11.5^a^	9.8	11.0	8.0	9.8^a^	8.1	10.9	9.0
*Victim’s age years*^e^																
0–5	786	31.1	672	85.7	62	10.2^b^	15	1.6^b^	37	17.5^b^	624	30.5	90	41.9^c^	72	26.7
6–13	684	27.0	43	5.5	468	76.7^b^	103	11.2^b^	70	33.0^b^	541	26.5	58	27.0	85	31.5^b^
14–17	794	31.4	1	0.1	49	8.0^b^	700	75.8^b^	44	20.8^b^	685	33.5	42	19.5^b^	67	24.8^b^
< 18, age unknown	46	1.8	0	0.0	2	0.3	2	0.2	42	19.8^b^	26	1.3	4	1.9	16	5.9^b^
18 and older	194	7.7	62	7.9	27	4.4^b^	90	9.8	15	7.1	149	7.3	20	9.3	25	9.3
Unknown	25	1.0	6	0.8	2	0.3	13	1.4	4	1.9	19	0.9	1	0.5	5	1.9
*Victim's gender*^e^																
Male	1925	76.1	579	73.9	463	75.9	751	81.4^b^	132	62.3^b^	1758	86.0	46	21.4^b^	121	44.8^b^
Female	476	18.8	166	21.2	135	22.1	133	14.4^b^	42	19.8	266	13.0	166	77.2^b^	44	16.3
Unknown	128	5.1	39	5.0	12	2.0^b^	39	4.2	38	17.9^b^	20	1.0	3	1.4	105	38.9^b^

*SD* standard deviation

*Notes*: Columns contain numbers and percentages unless otherwise noted. Mean age of the shooters was 10.0 (SD = 5.5) and ranged from 1 to 17 years old. Means, standard deviations, and percentages are rounded to the 10th or 100th decimal place

^a^*P*-value < 0.05 from t-test for difference in means between this shooter age/gender group and shooters 0–5 years old/males

^b^*P*-value < 0.05 from chi-square test for difference in percentages between this shooter age/gender group and shooters 0–5 years old/males

^c^Other locations include, but are not limited to, buses, daycares, woods, communal areas of apartment buildings, abandoned homes, and mobile homes

^d^Other gun types include assault-style weapons (e.g., AR-15), homemade unserialized “ghost guns,” and black powder guns

^e^Based on total victims including those who were shot by themselves or another person. The sum of victims will add to more than the total number of incidents. The age of victims ranged from a 1-month-old baby boy to a 77-year-old man

*Source:* Everytown for Gun Safety Support Fund. #NotAnAccident Index, 2015–2021

The majority of shooters (81.4%, 1993) and victims (76.1%, 1925) were male (Table 1). Shootings most often occurred in or around homes (71.4%, 1749), with shootings by younger children more likely to occur at home (82.4%, 0–5 years; 59.1%, 14–17 years; *p* < 0.01). Handguns were accessed in at least 53.0% (1298) of shootings. The weapon type is unknown for 37.8% of incidents. Excluding unknowns from the weapons total increases the proportion of handguns accessed to 85.3% (1298/1522).

Overall, children were as likely to shoot themselves (48.8%, 1195) as they were to shoot another person (47.2%, 1156) (Table 1). However, this varied by age group. Nearly three-quarters of children ages 0–5 years shot themselves (73.7%), whereas more than half of children and teens ages 6–13 and 14–17 shot someone else (54.7% and 58.3%, respectively; *p* < 0.01).

While inclusion criteria limits shooters in the dataset to be 17 years and younger, the age of victims ranged from a 1-month-old baby boy to a 77-year-old man (Table 1). The mean age of shooters was 10.0 years (standard deviation [SD] 5.5) and the mean age of victims was 10.9 years (SD 8.1). However, the majority of victims were under 18 years old (91.3%, 2310) (this number includes 46 victims who were under 18 years old, but their age group is unknown). A greater proportion of shooters were between the ages of 14 and 17 (36.5%, 894) than the share of toddlers ages 0–5 (31.2%, 763), whereas 14- to 17-year-olds (31.4%, 794) and 0- to 5-year-olds (31.1%, 786) were equally likely to be victims.

**Fig. 1:**
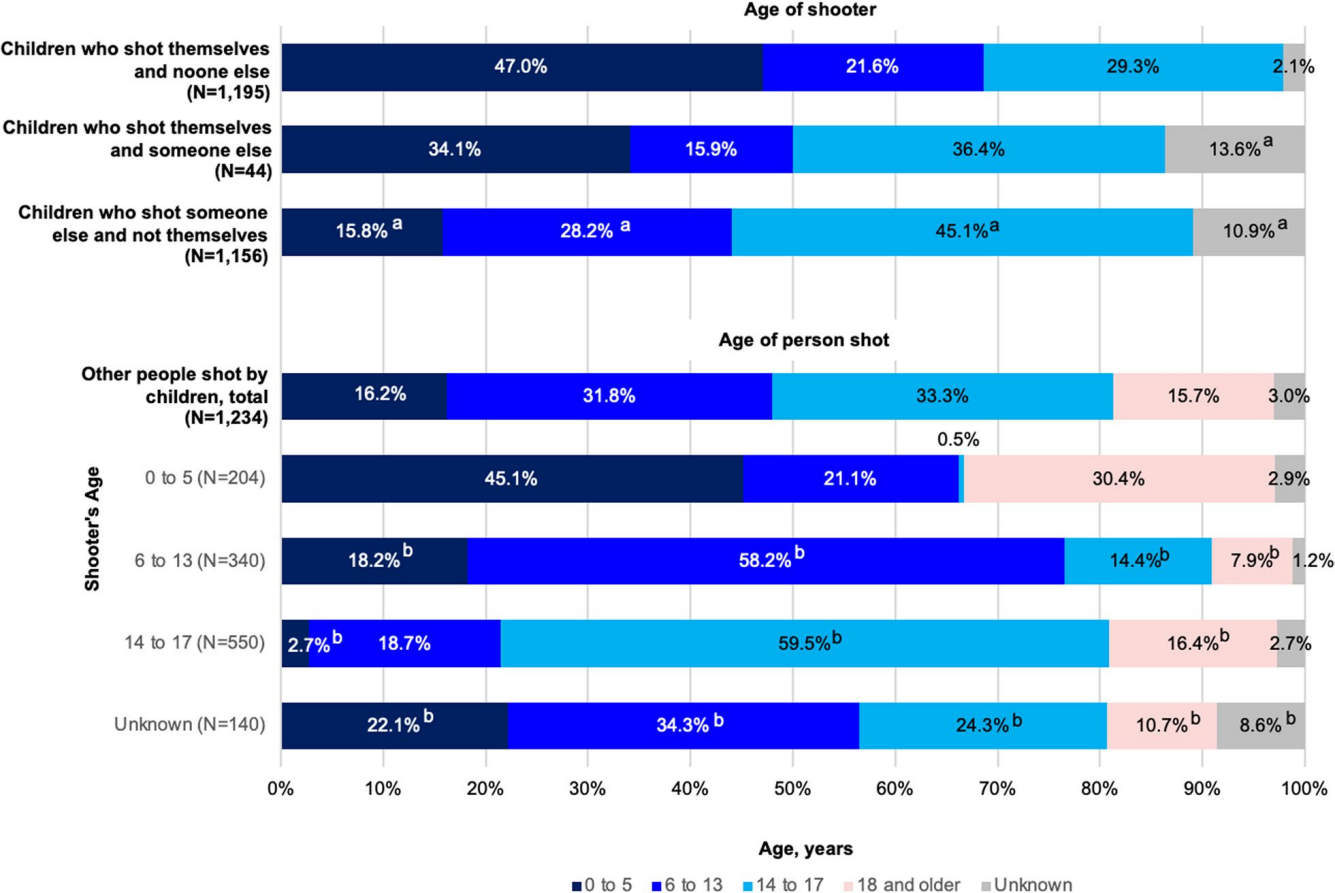
Age of person shot in unintentional shootings by children, by shooting type and age of shooter and victim, 2015–2021. ^a^*P*-value < 0.05 from chi-square test comparing this percentage to the percentage of children in this age category who shot themselves and no one else. ^b^*P*-value < 0.05 from chi-square test comparing this percentage to the percentage of victims in this age category injured by 0 to 5 year-old shooters. *Source*: Everytown for Gun Safety Support Fund. #NotAnAccident Index, 2015–2021.

When children shoot another person, they are most likely to shoot someone within their same age group (Fig. 1). However, the percentage of victims in a different age group varied by age of the shooter. Among shooters ages 0–5 years old, adults 18 years and older make up the next largest proportion of victims (30.4%). In contrast, 6- to 13-year-olds are next most likely to shoot children 0–5 (18.2%), and teenagers 14–17 are second most likely to shoot 6- to 13-year-olds (18.7%).

### Trends in unintentional shootings by children

**Fig. 2:**
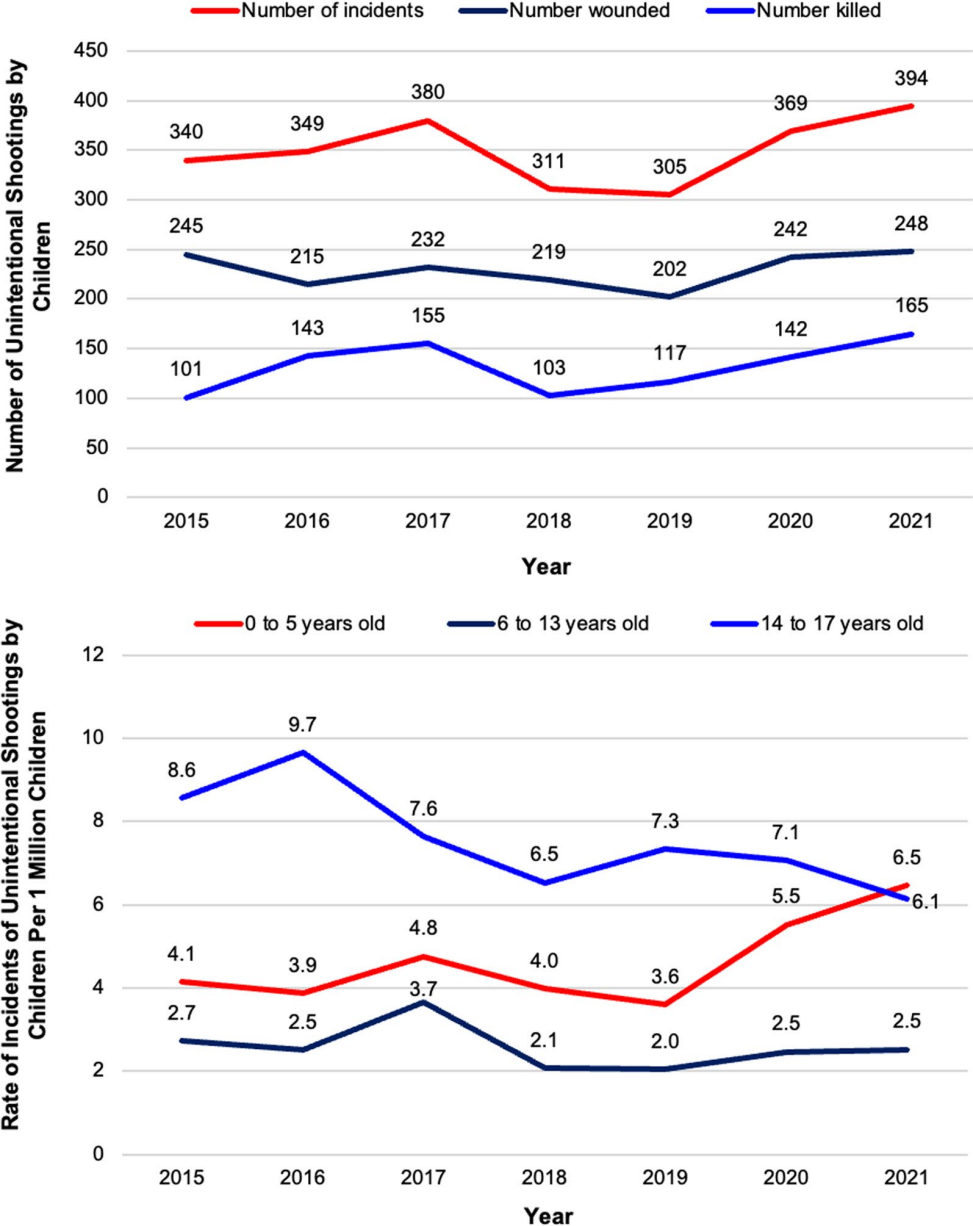
Unintentional shootings by children overall and rate per 1 million US children by age group of shooter, 2015–2021. Source: Everytown for Gun Safety Support Fund. #NotAnAccident Index, 2015–2021

The number of incidents per year ranged from 305 incidents in 2019 to 394 in 2021 (349.7 average incidents per year) (Fig. 2). The number of people shot and killed ranged from 101 in 2015 to 165 in 2021 (132.3 average per year), and the number of people shot and wounded ranged from 202 in 2019 to 248 in 2021 (229.0 average per year).

Unintentional shootings by children incidents, deaths, and injuries increased during 2020 and 2021, coinciding with the COVID-19 pandemic (Fig. 2). Comparing incidents during March to December 2020 with the same period in 2019, incidents increased 23.6% (314 vs 254), deaths increased 33.3% (128 vs 96), injuries increased 17.1% (199 vs 170), and total victims increased 22.9% (327 vs 266). Comparing incidents during January to December 2021 with the same period in 2020, incidents increased 6.8% (394 vs 369), deaths increased 16.2% (165 vs 142), injuries increased 2.5% (248 vs 242), and total victims increased 7.6% (413 vs 384).

The recent increase in the incidents of unintentional shootings by children has been driven by children ages 5 years and younger (Fig. 2). The rate of unintentional shooting incidents by children ages 0–5 years was 3.6 per 1 million US children in 2019 and rose to 6.5 in 2021—an 80.6% increase. During the same period, the rate declined among teenagers 14–17 years old, dropping from 7.3 to 6.1—a 16.4% decrease.

### Unintentional shootings by children by state

**Fig. 3:**
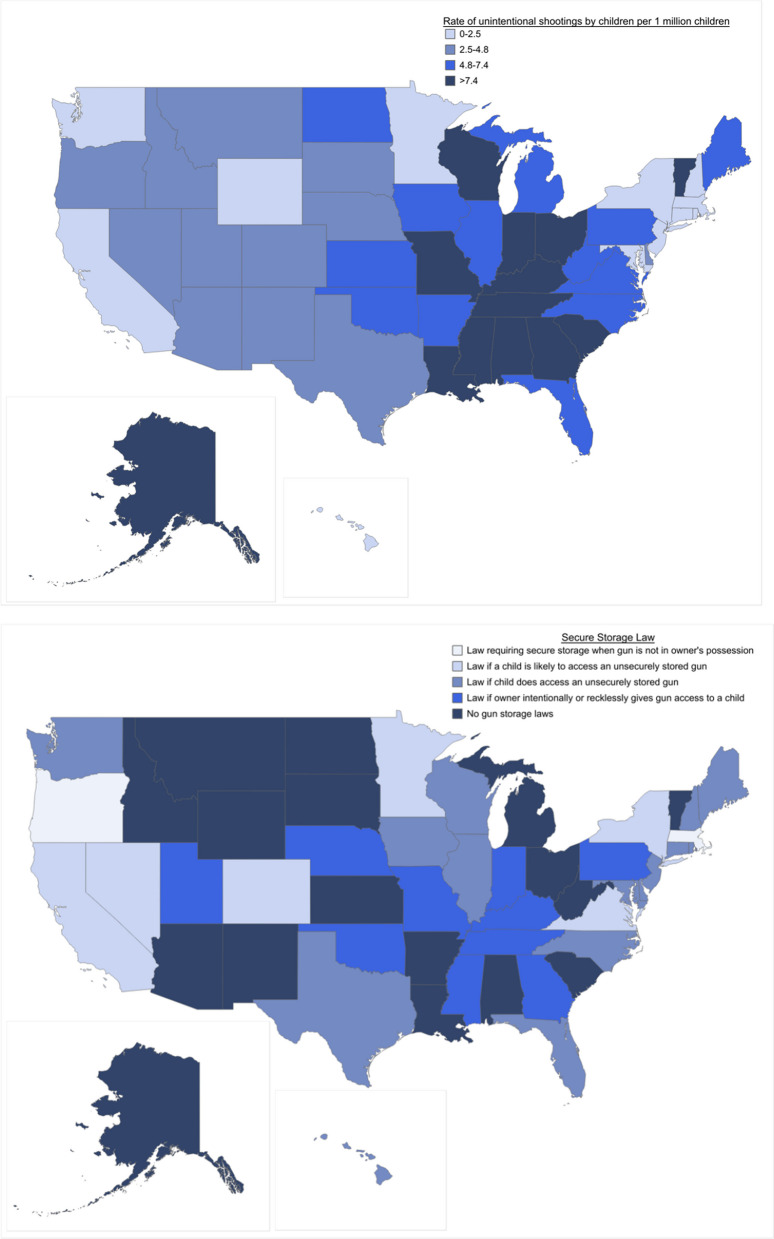
State rates of unintentional shootings by children and state secure storage laws. *Note* Represents laws effective as of 2021. Six states strengthened laws during the study period (CO, ME, NV, NY, OR, WA). *Source*: Everytown for Gun Safety Support Fund. #NotAnAccident Index, 2015–2021.

Unintentional shootings by children varied by state (Fig. 3). The 10 states with the highest rates per 1 million children were Louisiana (15.2), Mississippi (13.3), Alaska (11.7), Tennessee (11.1), Missouri (10.6), South Carolina (9.9), Alabama (9.4), Kentucky (8.8), Georgia (8.3), and Ohio (8.3). The 10 states where incidents were rare or never happened during this time period were Hawaii (0.0), Rhode Island (0.0), New Hampshire (0.6), Massachusetts (0.6), California (1.1), Wyoming (1.1), New Jersey (1.1), New York (1.3), Washington (1.8), and Connecticut (1.9). In the 10 states with the highest incident rates, the rates were on average 11 times higher than in the 10 states with the lowest rates (10.7 and 0.9, respectively).

### State-level secure storage laws and unintentional shootings by children

Compared with states with no secure storage laws, rates of unintentional shootings by children were 72% lower in states requiring secure storage when the gun is not in the owner’s possession (RR: 0.28, 95% CI 0.15–0.54, *p* < 0.01), 41% lower in states with laws that apply when a child is likely to access a gun (RR: 0.59, 95% CI 0.45–0.78, *p* < 0.01), and 24% lower in states with laws that apply when a child does access a gun (RR: 0.76, 95% CI 0.61–0.94, *p* = 0.01) (Table 2). States with laws that apply only if the gun owner intentionally or recklessly gives a child access to a gun had similar rates of unintentional shootings by children as those without any laws.

**Table 2:** The relationship between state-level secure gun storage laws and the rate of unintentional shootings by children

Storage law	Rate ratio (95% CI)	*p*-value
No gun storage laws	[Ref]	[Ref]
Law if gun owner intentionally or recklessly gives gun access to a child	1.06 (0.85, 1.31)	0.62
Law if child does access an unsecurely stored gun	0.76 (0.61, 0.94)	0.01
Law if child is likely to access an unsecurely stored gun	0.59 (0.45, 0.78)	< .01
Law requiring secure storage when not in owner’s possession	0.28 (0.15, 0.54)	< .01

*CI* confidence interval

*Note* Models controlled for the percentage of households with a firearm in the state and for the following population characteristics averaged across counties in the state: percentage of population with less than a high school education, percentage of the population that is male, unemployment rate, homicide death rate, suicide death rate, population density, mean household income. Additionally, the models included a fixed effect for census division and year

*Source:* Everytown for Gun Safety Support Fund. #NotAnAccident Index, 2015–2021

## Discussion

This study uses a unique data set that tracks unintentional shootings by children, including victims of all ages. We found that unintentional shootings by children are a persistent problem across the United States with incidents occurring in 48 of 50 states and Washington, DC, between 2015 and 2021. These shootings impacted more than 2000 families during this time period.

Many findings of this study are consistent with prior studies of unintentional injuries. Like prior studies, we found that the majority of shooters and victims were males (Hemenway and Solnick 2015; Solnick and Hemenway 2019; Price et al. 2021; Lee and Harris 1993; Sinauer et al. 1996) and that incidents most often occurred in or around homes (Lee and Harris 1993; Li et al. 1996; Centers for Disease Control and Prevention 2019), and involved handguns (Sinauer et al. 1996; Martin et al. 1991; Ismach et al. 2003). Our findings differ from prior research in one area. Hemenway and Solnick found that cases where children unintentionally shoot adults are rare, particularly among young shooters (Hemenway and Solnick 2015). This analysis shows 7.7% of incidents involved a victim age 18 or older, and among incidents where the shooter shot someone else, 30.4% of victims shot by 0- to 5-year-olds were 18 years or older.

Researchers have been documenting the rise in gun violence during the COVID-19 pandemic (Cohen et al. 2021; Peña and Jena 2022; Donnelly et al. 2022; Ssentongo et al. 2021). Amidst a surge in gun sales in 2020 and 2021 (Nass and Barton 2022), this study shows an increase in unintentional shootings by children in 2020 and 2021 when children were spending more time at home, particularly during the first year of the pandemic.

Our analysis of the relationship between unintentional shooting incidents by children and four types of laws that hold gun owners or other adults liable for failing to prevent firearm access by children suggests the most restrictive laws were most protective and the laws became less protective the weaker they were. Previous researchers have examined the impact of secure storage (commonly referred to as Child Access Prevention (CAP) laws) on rates of injury or death finding that these laws are associated with decreases in unintentional firearm injuries and deaths among children (Azad et al. 2020; Hamilton et al. 2018; RAND Corporation 2020). To our knowledge we are the first to look at the impact of these laws on rates of unintentional shootings by children. We are also the first to measure secure storage laws in this way, based on Everytown’s Gun Law Rankings (Everytown for Gun Safety Support Fund 2022) and additional legal research, which include newly adopted and most restrictive laws requiring secure storage when the gun is not in the owner’s immediate possession. Our findings that states with the strongest laws related to preventing firearm access by children have the lowest rates of unintentional shootings by children, complement prior research and provide more evidence to support the passage of secure storage laws. However, this finding should be interpreted with the context that causality cannot be determined. We could not account for awareness of the laws and their influence on behavior change, or enforcement of these laws in our models. Research is needed on the enforcement of these laws and any racial disparities in charging decisions and convictions.

Regardless of whether a state has a secure storage law, this study suggests that adults need to take more action to ensure that guns are stored securely and inaccessible to children to prevent more of these tragedies. Research strongly links secure storage practices with decreased risk for firearm injury (Violano et al. 2018). One prior study found that households that locked both firearms and ammunition were associated with an 85 percent lower risk of unintentional firearm injuries among children, compared to those that locked neither (Grossman et al. 2005). Another study estimated that if half of households with children that contain at least one unlocked gun switched to locking all their guns, one-third of youth gun suicides and unintentional deaths could be prevented, saving an estimated 251 lives in a single year (Monuteaux et al. 2019).

Media reports of unintentional shootings by children reveal that children are accessing loaded guns left unsecured in closets and nightstand drawers, in backpacks and purses, underneath couch cushions, beds, and car seats, or just left out in plain view (Harris 2022; WLOS 2022; Garger 2021; Schnur 2022; Komer 2022; Edwards 2022; La Plante 2021; Kenton 2021). Efforts to teach children to stay away from guns have proven to be ineffective at modifying gun playing behavior (Holly et al. 2019), making it imperative to invest in prevention efforts focused on adults. Experts recommend that guns be stored unloaded, locked, and separate from ammunition (Lee et al. 2022; U.S. Government Accountability Office 2017).

Public awareness campaigns to promote public health have been effective at producing behavior change (Wakefield 2010; Akbari et al. 2021; Tan et al. 2022). While evaluation of such campaigns in the gun safety field is scant, several programs have been implemented, including the national Be SMART and End Family Fire campaigns. Emerging research on these two programs in clinical settings shows promising results (Silver et al. 2021; Gastineau et al. 2021; Clary et al. 2020; Hoops et al. 2021). The Be SMART campaign, implemented by volunteers for Moms Demand Action for Gun Sense in America, seeks to normalize conversations about secure gun storage, encouraging adults to follow five steps: Secure all guns in their homes and vehicles; Model responsible behavior around guns; Ask about the presence of unsecured guns in other homes; Recognize the role of guns in suicide; and Tell your peers to be SMART (Everytown for Gun Safety Support Fund 2023; Thomas et al. 2019). End Family Fire, a partnership between Brady and the Ad Council, is a national advertising campaign that promotes responsible gun ownership and encourages secure gun storage (Brady United Against Gun Violence and AD Council 2023). Both of these programs are adaptable to be implemented by various stakeholders including doctors (Silver et al. 2021; Gastineau et al. 2021; Clary et al. 2020; Hoops et al. 2021), educators (Sawchuk 2021), government officials (Everytown for Gun Safety Action Fund 2020), and law enforcement (Woodland Park Police Department 2021), among others. Further research on the effectiveness of public education on secure firearm storage in community settings is needed.

The American Academy of Pediatrics recommends that pediatricians address firearm safety when counseling parents. Studies show these conversations can influence gun storage practices (Barkin et al. 2008). Clinician counseling on firearms has been shown to be particularly effective when combined with the distribution of free gun locks (Barkin et al. 2008; Rowhani-Rahbar et al. 2016; Carbone et al. 2005).

The main limitation of this study is the dataset is compiled from media reports. The number of unintentional shootings by children in this dataset is likely an underestimate because, as research has shown, the media fails to cover a significant portion of shooting incidents (Kaufman et al. 2020; Marvel et al. 2018). The extent to which incidents are undercounted more often in states with strong or weak secure storage laws is unknown and may bias the regression results in either direction. Importantly, even when these incidents are covered, reporters often fail to include important details that could help inform prevention efforts (Marvel et al. 2018), such as the type of weapon, who owned the gun, and how the gun was stored. However, a strength of this study is that it accurately determines the intent of the shooting as unintentional based on multiple reviews. In contrast, researchers have consistently found that counts of unintentional deaths in vital statistic registries are inaccurate, and often are misclassified as homicides, particularly when a young person is shot by another person (Schaechter et al. 2003; Barber and Hemenway 2011; Luo and McIntire 2013; Hemenway and Solnick 2015; Solnick and Hemenway 2019). The fatal gun injuries in this paper correspond to 26.5% (926/3498) of all unintentional gun deaths and 118.8% (847/713) of unintentional gun deaths among children 17 years and younger from 2015 to 2021 reported by the CDC (Centers for Disease Control and Prevention 2021, 2023).

Two important considerations enrich the discussion of our research. Careful selection of control variables will be important for future studies because it is unclear whether state policies are reflective of gun culture and thus measurable and unmeasurable confounding factors are related to both secure storage laws and unintentional shootings by children. Additionally, state policies may influence gun culture and therefore secure storage behavior, thereby decreasing these events. Furthermore, six states changed secure storage policies during the study time frame, most too late to evaluate the policy effects using a more sophisticated longitudinal design. Future research must explore these effects.

## Conclusions

Unintentional shootings by children are on the rise, particularly among children 0–5 years old, but are preventable tragedies. Our results show that secure firearm storage policies are strongly correlated with lower rates of unintentional shootings by children. Firearm storage policies, practices, and education efforts are needed to ensure guns are kept secured and inaccessible to children.

## Methods

### Data source and study population

Demographic and injury data from 1/1/2015–12/31/2021 were extracted from Everytown’s #NotAnAccident Index (Everytown for Gun Safety Support Fund 2023). The #NotAnAccident Index contains media-report data of unintentional shooting incidents committed by children 17 years and younger in the US that resulted in gunshot injury or death of themselves or someone else of any age. News articles are systematically flagged through Google Alerts using a 15-word search string. Articles are reviewed and checked against inclusion criteria. Google searches are conducted to obtain additional news articles to check for the accuracy of reporting and gather additional details. The incident details are then coded by one researcher and are vetted by a second researcher and uploaded weekly. Data is reliability and validity tested quarterly by a third researcher comparing entries to incidents contained in the Gun Violence Archive’s repository of gun-related incidents compiled from over 7500 media, law enforcement, commercial, and government sources daily (Gun Violence Archive 2023).

In cases of self-inflicted gun injury or gun death, if the article does not include a clear determination of intent (e.g., by law enforcement) two researchers will review available information and make a determination on whether the shooting circumstances indicate if it was unintentional. If circumstances suggest the shooting was intentional or a determination could not be made, the incident is excluded from the database.

Additionally, state population data on children under 18 were obtained from the US Census Bureau (U.S. Census Bureau 2023), the state-level household firearm ownership rate was obtained from the RAND Corporation (Schell et al. 2020), and other population characteristics were obtained from the Agency for Healthcare Research and Quality Social Determinants of Health Database (Agency for Healthcare Research and Quality 2023).

### Variables

We examine characteristics of shooting incidents by children. The #NotAnAccident Index contains data on date, locality (city, state), location (home, car, public, etc.), victim and shooter demographics (age and gender), shooting type (shot self, shot others, shot self and others, unknown), victim outcomes (death or injury), firearm type (handgun, shotgun, rifle, etc.), and narrative incident summaries.

We also examine incident rates aggregated to the state level in each year. In addition to the incident rate, we examined secure firearm storage laws, which create liability for gun owners and other adults if a gun is not securely stored. Using Everytown’s Gun Law Rankings (Everytown for Gun Safety Support Fund 2022) and additional legal research, we grouped states into five categories based on the strictness of their laws during the study period, from most restrictive to least: 1. Law applies any time the gun is not in the owner’s immediate control (2 states as of 2021); 2. Law applies if a child may or is likely to access an unsecured gun (6 states and Washington, DC); 3. Law applies if a child does access an unsecured gun (15 states); 4. Law applies if the gun owner intentionally or recklessly gives a child access to a gun (10 states); and 5. No law (17 states). Six states changed status during the study period. Their storage law status in each year reflects whether the law was in effect in that year.

Finally, state population characteristics were averaged across counties in the state in each year: percentage of the population with less than a high school education, percentage of the population that is male, unemployment rate, population density, mean household income, homicide death rate, and suicide death rate. These variables, as well as the state-level household firearm ownership rate, were controls in the regression analysis described below.

### Analysis

In descriptive analyses, first, we examined characteristics of shooting incidents by children overall, and by age group and gender of the shooter. Second, we examined the number of shooters and victims by age group. Pairwise comparisons across age groups and gender were made using chi-square and t-tests. Third, we examined rates of unintentional shooting incidents by children by age group of the shooter from 2015 to 2021, which were calculated using state of occurrence and US Census data.

In addition, we used negative binomial regression models to examine whether rates of unintentional shootings by children are higher in areas with no or weak secure storage laws. In this analysis of a state*year level data set, the primary independent variable was the state’s secure storage law in each year using the five categories described above, where having no law was the reference group. The outcome was the number of incidents offset by the population of children under 18 years old. Models controlled for the percentage of the population with less than a high school education, percentage of the population that is male, unemployment rate, population density, mean household income, homicide death rate, suicide death rate, and the percentage of households with a firearm. Additionally, the models included a fixed effect for census division and year. Analyses were performed in STATA version 17.0.

## Data Availability

The dataset analyzed to generate the descriptive statistics in this study are available to download from the Everytown for Gun Safety Support Fund at https://everytownresearch.org/maps/notanaccident/. The dataset generated to conduct the analysis of incidents and state-level secure storage laws are available from the corresponding author on reasonable request.

## Abbreviations

CAP law: Child access prevention law
CDC: Centers for disease control and prevention
Everytown: Everytown for gun safety support fund
NVDRS: National violent death reporting system
NFR-CRS: National fatality review case reporting system

## References

[CR1] 2-year-old child accidentally shoots himself in Mitchell County, sheriff says. WLOS. 2022 Jan 5. https://wlos.com/news/local/2-year-old-child-accidently-shoots-himself-in-mitchell-county-sheriff-says.

[CR2] Agency for Healthcare Research and Quality. Social determinants of health database. Accessed 2023 Feb 1. https://www.ahrq.gov/sdoh/data-analytics/sdoh-data.html.

[CR3] Akbari M, Lankarani KB, Tabrizi R, Heydari ST, Vali M, Motevalian SA (2021). The effectiveness of mass media campaigns in increasing the use of seat belts: a systematic review. Traffic Inj Prev.

[CR4] Azad HA, Monuteaux MC, Rees CA, Siegel M, Mannix R, Lee LK (2020). Child access prevention firearm laws and firearm fatalities among children aged 0 to 14 years, 1991–2016. JAMA Pediatr.

[CR5] Barber C, Hemenway D (2011). Too many or too few unintentional firearm deaths in official US mortality data?. Accid Anal Prev.

[CR6] Barkin SL, Finch SA, Ip EH, Scheindlin B, Craig JA, Steffes J (2008). Is office-based counseling about media use, timeouts, and firearm storage effective? Results from a cluster-randomized, controlled trial. Pediatrics.

[CR7] Baxley F, Miller M (2006). Parental misperceptions about children and firearms. Arch Pediatr Adolesc Med.

[CR8] Bleyer A, Siegel SE, Thomas CR (2021). Increasing rate of unintentional firearm deaths in youngest Americans: firearm prevalence and Covid-19 pandemic implication. J Natl Med Assoc.

[CR9] Brady United Against Gun Violence and AD Council. End Family Fire. Accessed 2023 Feb 1. https://www.endfamilyfire.org/.

[CR10] Carbone PS, Clemens CJ, Ball TM (2005). Effectiveness of gun-safety counseling and a gun lock giveaway in a Hispanic community. Arch Pediatr Adolesc Med.

[CR11] Centers for Disease Control and Prevention, National Center for Health Statistics. National Vital Statistics System, Mortality 1999–2020 on CDC WONDER Online Database, released in 2021. Data are from the Multiple Cause of Death Files, 2015–2020. Accessed 2023 Feb 2. https://wonder.cdc.gov/ucd-icd10.html.

[CR12] Centers for Disease Control and Prevention, National Center for Health Statistics. National Vital Statistics System, Mortality 2018–2021 on CDC WONDER Online Database, released in 2023. Data are from the Multiple Cause of Death Files, 2021. Underlying Cause of Death, Injury Mechanism & All Other Leading Causes, among ages 1 to 17. Accessed 2023 Feb 2. Available from: http://wonder.cdc.gov/ucd-icd10-expanded.html.

[CR13] Centers for Disease Control and Prevention, National Center for Health Statistics. National Vital Statistics System, Mortality 2018–2021 on CDC WONDER Online Database, released in 2023. Data are from the Multiple Cause of Death Files, 2021. Accessed 2023 Feb 2. http://wonder.cdc.gov/ucd-icd10-expanded.html.

[CR14] Centers for Disease Control and Prevention, National Center for Injury Prevention and Control. National Violent Death Reporting System (NVDRS) on CDC WISQARS Online Database. Data from 2019, unintentional firearm deaths among ages 0 to 17. Accessed 2022 Aug 30. Available from: https://wisqars.cdc.gov/nvdrs/.

[CR15] Clary C, Lambarth L, Kaushik R (2020). Locked and (un)-loaded discussions: a pediatric resident safe firearm storage counseling curriculum. MedEdPORTAL.

[CR16] Cohen JS, Donnelly K, Patel SJ, Badolato GM, Boyle MD, McCarter R (2021). Firearm injuries involving young children in the United States during the COVID-19 pandemic. Pediatrics.

[CR17] Crifasi CK, Doucette ML, McGinty EE, Webster DW, Barry CL (2018). Storage practices of us gun owners in 2016. Am J Public Health.

[CR18] Donnelly MR, Grigoria A, Swentek L, Arora J, Kuza CM, Inaba K (2022). Firearm violence against children in the United States: trends in the wake of the COVID-19 pandemic. J Trauma Acute Care Surg.

[CR19] Edwards J. A mother smoked marijuana in the front seat, sheriff says. In the back, her 4-year-old found a gun and shot himself. Washington Post. 2022 Feb 2. https://www.washingtonpost.com/nation/2022/02/02/louisiana-4-year-old-accidental-shooting-mother-smoking-marijuana/.

[CR20] Everytown for Gun Safety Support Fund. #NotAnAccident index. Accessed 2023 Jan 31. https://everytownresearch.org/maps/notanaccident/.

[CR21] Everytown for Gun Safety Support Fund. Gun law rankings: which states have child access and/or secure storage laws? 2022. https://everytownresearch.org/rankings/law/secure-storage-or-child-access-prevention-required/.

[CR22] Everytown for Gun Safety Support Fund. Be SMART. Accessed 2023 Feb 2. https://besmartforkids.org/.

[CR23] Everytown for Gun Safety Action Fund. As gun purchases skyrocket during pandemic, Everytown for Gun Safety, Moms Demand Action unveil “Be SMART” PSA on secure gun storage. Press release. 2020 Apr 23. https://www.everytown.org/press/as-gun-purchases-skyrocket-during-pandemic-everytown-for-gun-safety-moms-demand-action-unveil-be-smart-psa-on-secure-gun-storage/.

[CR24] Garger K. Charges filed against father of toddler who fatally shot mom on Zoom call. New York Post. 2021 Oct 13. https://nypost.com/2021/10/13/charges-filed-against-veondre-avery-after-toddler-fatally-shot-mom-shamaya-lynn/.

[CR25] Gastineau KAB, Stegall CL, Lowrey LK, Giourgas BK, Andrews AL (2021). Improving the frequency and documentation of gun safety counseling in a resident primary care clinic. Acad Pediatr.

[CR26] Grossman DC, Mueller BA, Reidy C, Dowd D, Villaveces A, Prodzinski J (2005). Gun storage practices and risk of youth suicide and unintentional firearm injuries. JAMA.

[CR27] Gun Violence Archive. About us. Accessed 2023 Feb 2. https://www.gunviolencearchive.org/.

[CR28] Hamilton EC, Miller CC, Cox CS, Lally KP, Austin MP (2018). Variability of child access prevention laws and pediatric firearm injuries. J Trauma Acute Care Surg.

[CR29] Harris C. Boy, 8, was playing with dad's gun when a 1-year-old girl was killed and her sister, 2, was injured: sheriff. People. 2022 Jun 29. https://people.com/crime/boy-8-playing-dads-gun-killed-1-year-old-girl-injured-2-year-old-sister/.

[CR30] Hemenway D, Solnick SJ (2015). Children and unintentional firearm death. Inj Epidemiol.

[CR31] Holly C, Porter S, Kamienski M, Lim A (2019). School-based and community-based gun safety educational strategies for injury prevention. Health Promot Pract.

[CR32] Hoops KEM, Hernandez E, Ziegfeld S, Nasr I, Crifasi C (2021). Evaluating the use of a pamphlet as an educational tool to improve safe firearm storage in the home. Clin Pediatr.

[CR33] Ismach RB, Reza A, Ary R, Sampson TR, Bartolomeos K, Kellermann AL (2003). Unintended shootings in a large metropolitan area: an incident-based analysis. Ann Emerg Med.

[CR34] Kaufman EJ, Passman JE, Jacoby SF, Holena DN, Seamon MJ, MacMillan J (2020). Making the news: victim characteristics associated with media reporting on firearm injury. Prev Med.

[CR35] Kenton L. North Carolina mom of five, 25, is killed after one of her own children found a loaded gun in her purse and accidentally shot her as three of her other kids watched on. Daily Mail. 2021 Feb 17. https://www.dailymail.co.uk/news/article-9272439/Mom-five-25-killed-one-children-gun-purse-shot-her.html.

[CR36] Komer D. 2-year-old shoots himself with gun on Detroit’s east side, expected to recover. Fox 2 Detroit. 2022 Feb 7. https://www.fox2detroit.com/news/2-year-old-shoots-himself-with-gun-on-detroits-east-side.

[CR37] La Plante M. Port St. Lucie police locate gun connected to June 11 shooting of a 2-year-old child. Treasure Coast Newspapers. 2021 Jun 22. https://www.tcpalm.com/story/news/crime/martin-county/2021/06/22/gun-connected-port-st-lucie-sho.

[CR38] Lee LK, Fleegler EW, Goyal MK, Doh KF, Laraque-Arena D, Hoffman BD (2022). Firearm-related injuries and deaths in children and youth: injury prevention and harm reduction. Pediatrics.

[CR39] Lee RK, Harris MJ (1993). Unintentional firearm injuries: the price of protection. Am J Prev Med.

[CR40] Li G, Baker SP, DiScala C, Fowler C, Ling J, Kelen GD (1996). Factors associated with the intent of firearm-related injuries in pediatric trauma patients. Arch Pediatr Adolesc Med.

[CR41] Luo M, McIntire M. Children and guns: the hidden toll. New York Times. 2013 Sep 28; Sect. A:1. https://www.nytimes.com/2013/09/29/us/children-and-guns-the-hidden-toll.html.

[CR42] Martin JR, Sklar DP, McFeeley P (1991). Accidental firearm fatalities among New Mexico children. Ann Emerg Med.

[CR43] Marvel D, Mejia P, Nixon L, Dorfman L. Issue 25: More than mass shootings: gun violence narratives in California news. 2018 Jun 18. Berkley, CA: Berkley Media Studies Group. http://www.bmsg.org/resources/publications/gun-suicide-community-domestic-violence-news-narratives-california/.

[CR44] Miller M, Azrael D (2022). Firearm storage in US households with children: findings from the 2021 national firearm survey. JAMA Netw Open.

[CR45] Monuteaux MC, Azrael D, Miller M (2019). Association of increased safe household firearm storage with firearm suicide and unintentional death among US youths. JAMA Pediatr.

[CR46] Nass D, Barton C. How many guns did Americans buy last month? The Trace. 2022 Nov 8. https://www.thetrace.org/2020/08/gun-sales-estimates/.

[CR47] Peña P, Jena A (2022). Child deaths by gun violence in the US during the COVID-19 pandemic. JAMA Netw Open.

[CR48] Price JH, Khubchandani J, Foh EP (2021). Unintentional firearm mortality in African–American youths, 2010–2019. J Natl Med Assoc.

[CR49] RAND Corporation. Effects of child-access prevention laws on unintentional injuries and deaths. 2020 Apr 22. https://www.rand.org/research/gun-policy/analysis/child-access-prevention/unintentional-injuries.html.

[CR50] Rowhani-Rahbar A, Simonetti JA, Rivara FP (2016). Effectiveness of interventions to promote safe firearm storage. Epidemiol Rev.

[CR51] Salhi C, Azrael D, Miller M (2021). Parent and adolescent reports of adolescent access to household firearms in the United States. JAMA Netw Open.

[CR52] Sawchuk S. More schools are reminding parents to secure their guns. Education Week. 2021 Dec 8. https://www.edweek.org/leadership/more-schools-are-reminding-parents-to-secure-their-guns/2021/12.

[CR53] Schaechter J, Duran I, DeMarchena J, Lemard G, Villar ME (2003). Are ‘accidental’ gun deaths as rare as they seem? A comparison of medical examiner manner of death coding with an intent-based classification approach. Pediatrics.

[CR54] Schell TL, Peterson S, Vegetabile BG, Scherling A, Smart R, Morral AR (2020). State-level estimates of household firearm ownership.

[CR55] Schnur S. Woman sues ex-husband after fatal shooting of 6-year-old girl. Las Vegas Review-Journal. 2022 Nov 18. https://www.reviewjournal.com/crime/courts/woman-sues-ex-husband-after-fatal-shooting-of-6-year-old-girl-2678963/.

[CR56] Silver AH, Azzarone G, Dodson N, Curle M, Eisenberg R, Kim M, O’Connor K (2021). A randomized controlled trial for parents of hospitalized children: keeping kids safe from guns. Hosp Pediatr.

[CR57] Simonetti JA, Mackelprang JL, Rowhani-Rahbar A, Zatzick D, Rivara FP (2015). Psychiatric comorbidity, suicidality, and in-home firearm access among a nationally representative sample of adolescents. JAMA Psychiat.

[CR58] Sinauer N, Annest JL, Mercy JA (1996). Unintentional, nonfatal firearm-related injuries: a preventable public health burden. JAMA.

[CR59] Solnick SJ, Hemenway D (2019). Unintentional firearm deaths in the United States, 2005–2015. Inj Epidemiol.

[CR60] Ssentongo P, Frontier C, Ssentongo AE, Advani S, Heilbrunn ES, Hazelton JP (2021). Gun violence incidence during the COVID-19 pandemic is higher than before the pandemic in the United States. Sci Rep.

[CR61] Tan J, Ramazanu S, Liaw SY, Chua WL (2022). Effectiveness of public education campaigns for stroke symptom recognition and response in non-elderly adults: a systematic review and meta-analysis. J Stroke Cerebrovasc Dis.

[CR62] Thomas J, Chappell-Deckert J, Thomas S (2019). The Be SMART initiative: educating communities on safe gun practices. J Pub Health Issue Pract.

[CR63] Trigylidas TE, Lichenstein R, Schnitzer PG, Dykstra HK, Badolato GM, Goyal MK (2021). Firearm deaths among youth in the United States, 2007–2016. Pediatrics.

[CR64] Trigylidas TE, Schnitzer PG, Dykstra HK, Lichenstein R (2019). Unintentional firearm deaths among youth in the United States. Ann Emerg Med.

[CR65] U.S. Government Accountability Office. Personal firearms: programs that promote safe storage and research on their effectiveness. 2017 Sep. Report No.:GAO-17-665. https://www.gao.gov/products/gao-17-665.

[CR66] U.S. Census Bureau. Table S0901: Children under 18 in households, 2015–2021. Accessed 2023 Feb 1. https://data.census.gov/.

[CR67] Violano P, Bonne S, Duncan T, Pappas P, Christmas AB, Dennis A (2018). Prevention of firearm injuries with gun safety devices and safe storage: an Eastern Association of the Surgery of trauma systematic review. J Trauma Acute Care Surg.

[CR68] Wakefield MA, Loke B, Hornik RC (2010). Use of mass media campaigns to change health behaviour. Lancet.

[CR69] Woodland Park Police Department, gun range support Be SMART safety initiative. Tap Into Passaic Valley. 2021 Mar 22. https://www.tapinto.net/towns/passaic-valley/sections/health-and-wellness/articles/woodland-park-police-department-gun-range-support-be-smart-safety-initiative/.

